# Emergence of Community-Associated Methicillin-Resistant *Staphylococcus aureus* Carriage in Children in Cambodia

**DOI:** 10.4269/ajtmh.2011.10-0300

**Published:** 2011-02-04

**Authors:** Emma K. Nickerson, Vanaporn Wuthiekanun, Varun Kumar, Premjit Amornchai, Nattavut Wongdeethai, Kheng Chheng, Narisara Chantratita, Hor Putchhat, Janjira Thaipadungpanit, Nicholas P. Day, Sharon J. Peacock

**Affiliations:** Mahidol-Oxford Tropical Medicine Research Unit, and Department of Microbiology and Immunology,Faculty of Tropical Medicine, Mahidol University, Bangkok, Thailand; Department of Infectious Diseases, and Department of Medicine, Addenbrooke's Hospital, Cambridge, United Kingdom; Angkor Hospital for Children, Siem Reap, Cambodia; Centre for Clinical Vaccinology and Tropical Medicine, Nuffield Department of Clinical Medicine, University of Oxford, Churchill Hospital, Oxford, United Kingdom

## Abstract

We previously described the first reported isolation of methicillin-resistant *Staphylococcus aureus* (MRSA) (a case series of pediatric community-associated MRSA infections) in Cambodia. We define the rate of pediatric MRSA carriage in the same population and characterize the associated bacterial genotypes by using pulsed-field gel electrophoresis and multilocus sequence typing. A prospective cohort study of MRSA carriage conducted over one month at the Angkor Hospital for Children, Siem Reap, Cambodia, identified MRSA carriage in 87 (3.5%) of 2,485 children who came to the outpatient department, and 6 (4.1%) of 145 inpatients, including at least two with cases of nosocomial acquisition. Genotyping of all 93 MRSA isolates resolved 5 genotypes. Most (91%) isolates were assigned to sequence type 834. Only 28 (32%) of 87 MRSA carriers identified in the outpatient department had no history of recent healthcare contact. The study findings have important implications for healthcare in a setting where diagnostic microbiology and access to antimicrobial drugs with efficacy against MRSA are limited.

## Introduction

Carriage and infection by community-associated methicillin-resistant *Staphylococcus aureus* (CA-MRSA) has become an important public health threat in developed countries,^1^–^3^ but there is a comparative lack of published information on the emergence of CA-MRSA in developing countries. Possible reasons for the latter include a time delay for highly transmissible CA-MRSA clones to spread from the developed to the developing world, an absence of the *in situ* emergence of CA-MRSA clones in developing world settings, or under-reporting associated with a paucity of diagnostic microbiology facilities. The efficiency of transmission observed in developed countries for CA-MRSA clones such as US300,^1^,^2^,^4^ combined with evidence that MRSA can arise in multiple genetic lineages^5^ argues against the idea that CA-MRSA clones have not emerged in the developing world. Furthermore, factors that have been linked to CA-MRSA emergence and infection in several remote settings^6^,^7^ are prevalent throughout the developing world. These factors include a high frequency of antimicrobial drug consumption, sub-standard living accommodation with over-crowding and lack of access to water for bathing, a high frequency of superficial staphylococcal skin sepsis in the community, and frequent scratches/insect bites or skin infestation with scabies.^6^,^7^ We propose that CA-MRSA is likely to have already arisen in multiple resource-poor settings, but that there is marked under-reporting of such events associated with a lack of microbiology facilities.

We work at the Angkor Hospital for Children (AHC), a non-governmental organization–funded children's hospital in Siem Reap, northwestern Cambodia. A diagnostic microbiology laboratory was introduced into this hospital in January 2006, and over the following 24 months, 17 children were identified with CA-MRSA infection, of which 11 were associated with skin and soft tissue infection and 6 were associated with invasive disease.^8^ This finding was the first description of MRSA isolation in Cambodia, a setting where diagnostic laboratory facilities are limited. We postulated that these findings were sporadic infections in persons who were MRSA carriers or contacts of carriers on the basis that cases were unrelated in time and involved children who were geographically dispersed.

Molecular characterization of these 17 isolates identified two independent MRSA clones; 15 isolates were defined as sequence type (ST) 834, staphylococcal cassette chromosome mec (SCC*mec*) type IV, Panton-Valentine leukocidin gene negative, and two isolates were defined as ST 121, SCC*mec* type V, Panton-Valentine leukocidin gene positive. ST 834 was isolated on three occasions during 2006 in Western Australia, and ST 121 is the most common clone of methicillin-susceptible *S. aureus* (MSSA) associated with community-acquired *S. aureus* infection in children coming to the AHC.^8^ The purpose of our study was to define the presence and rate of MRSA carriage in children in the same geographic region and identify the genotypes of the associated isolates.

## Methods and Materials

### Study setting and patients.

The study setting was the AHC, which provides free outpatient department (OPD) care to approximately 400 children per day, and maintains 50 inpatient beds spread across high-, medium-, and low-intensity care areas. The patient population is drawn from an unrestricted catchment area. MRSA carriage was defined in unselected consecutive children treated in OPD alone or admitted to the hospital during September 18, 2008–October 18, 2008, together with children who were already an inpatient at the study start date. Inclusion criteria were presentation to the AHC during the study period and a willingness to participate. There were no exclusion criteria. The study protocol was reviewed and approved by the Ethical Review Board of the AHC, and written informed consent was obtained from a parent or guardian of all children recruited into the study.

Children treated in OPD alone received a swab of both nares on one occasion, and patients admitted to the hospital had nasal, axillae, and throat swabs (or tracheal suction samples if ventilated) twice a week until discharge or death. Swabs were placed into a sterile bijou containing 1 mL of sterile phosphate-buffered saline and returned to the laboratory for processing within 2 hours. The distal end of the tubing from tracheal suction catheters was removed by using sterile scissors and placed into a universal container prior to transfer to the laboratory.

### MRSA isolation and identification.

Bijous were vortexed and 100 μL of swab fluid was spread plated onto a mannitol salt agar (MSA) plate containing cefoxitin at a concentration of 4 mg/L. The remainder of the fluid and the swab were added to 8 mL of mannitol salt broth containing 4 mg/L of cefoxitin and incubated for 48 hours at 37°C in air. Ten microliters was then spread onto an MSA plate. Suction tubing samples were vortexed and the sample fluid was centrifuged for 5 minutes at 2,500 rpm. Excess supernatant was removed to leave approximately 200 μL and the pellet was resuspended, after which 100 μL was plated onto an MSA plate, and the remainder was added to broth and processed as described above. All agar plates were incubated at 37°C in air and examined daily for 2 days at which time yellow colonies were characterized by using the catalase and coagulase tests and a commercial slide agglutination test (Staphaurex Plus; Oxoid, Basingstoke, United Kingdom).

Methicillin resistance was initially confirmed by using a cefoxitin disk (30 μg) on Mueller-Hinton agar. Susceptibilities of MRSA isolates to cefoxitin, chloramphenicol, ciprofloxacin, clindamycin, erythromycin, fusidic acid, gentamicin, mupirocin, netilmicin, penicillin, rifampicin, trimethoprim/sulfamethoxazole, tetracycline, and vancomycin were tested by using the disk diffusion method.^9^ Inducible clindamycin resistance was determined as described.^10^ Minimum inhibitory concentrations were defined to oxacillin and vancomycin by using E-test methods (AB Biodisk, Solna, Sweden). Isolates were stored at –80°C in trypticase soy broth containing 20% (v/v) glycerol.

### Data collection and definitions.

Data obtained on all children were age, area of residence, and previous healthcare exposure (hospital admission in the preceding 12 months or any attendance at an outpatient clinic in the preceding 6 months). Additional data collected on inpatients alone were hospital ward and date of admission and discharge. The criteria used to define CA-MRSA were adapted from those published by the Centers for Disease Control and Prevention, Atlanta, GA (http://www.cdc.gov/ncidod/dhqp/ar_mrsa_ca.html). MRSA was considered to be community-associated if recovered from children presenting to OPD or from inpatients within 48 hours of hospital admission who had no medical history of MRSA infection or colonization and had no healthcare contact (hospital admission during the preceding 12 months or any attendance at an outpatient clinic in the preceding 6 months). Some criteria listed by the Centers for Disease Control and Prevention (admission to a hospice or nursing facility, dialysis, and permanent indwelling catheters or medical devices) were not relevant to our setting.

### Bacterial genotyping.

All MRSA isolates were genotyped by using pulsed-field gel electrophoresis (PFGE) and *Sma I* as the restriction enzyme, as described.^11^ Two MRSA isolates belonging to each of the multilocus sequence typing (MLST)–defined clones ST 834 and MRSA ST 121, which were identified previously at AHC,^8^ were run on every gel as known controls. Banding patterns were analyzed by using BioNumerics software version 2.5 (Applied Maths, Sint-Martens-Latem, Belgium). Percent similarities were identified on a dendrogram derived from the unweighted pair group method using arithmetic averages and based on Dice coefficients. Band position tolerance and optimization were set at 1.25 and 0.5%, respectively. A similarity coefficient of 80% was selected to define clusters of pulsed-field gel types. At least two bacterial representatives of each pulsed-field gel type were further examined by using MLST (or a single isolate if this was the only representative of a given PFGE banding pattern type). Genomic DNA extraction was performed as described,^8^ and MLST was performed as described by Enright and others.^12^ Sequence type assignment was based on the sequence of the alleles at each locus of seven housekeeping genes by using the MLST database (www.mlst.net). The STs were assigned to their clonal complex using the program eBURST (http://eburst.mlst.net) and the entire MLST database.

## Results

### Prevalence of MRSA carriage.

A total of 2,630 children were recruited into the study, of whom 93 (3.5%) had MRSA. Antimicrobial susceptibility profiles for these 93 isolates are shown in Table 1.

#### Outpatient population.

A total of 2,485 patients with a median age of 3 years (interquartile range = 1 year 3 months to 6 years 7 months) were seen in OPD alone, of whom 87 (3.5%) of 2,485 had MRSA. There was no association between MRSA carriage and age (*P* = 0.13). Only 28 (32%) of 87 carriers fulfilled our definition for CA-MRSA carriage. The remaining 59 carriers had a history of previous healthcare exposure, although the source of MRSA acquisition in these cases could not be accurately determined. There was a significant difference in the presence of a history of healthcare exposure between MRSA carriers and non-carriers in this OPD population, (68% [59 of 87] versus 48% [1,163 of 2,398]), respectively (*P* < 0.001)). Hospital admission was significantly associated with carriage (8.5% carriers admitted versus 2.6% carriers not admitted; *P* < 0.001).

#### Inpatient population.

An additional 145 children with a median age of 2 years (interquartile range = 9 months to 8 years 9 months) were recruited from inpatient wards, of whom 6 (4.1%) carried MRSA. This organism was isolated from the nares of 5 of 6 patients; the sixth patient initially had a negative nasal swab but positive swabs from throat and axillae and subsequently became colonized in the nose. We considered whether MRSA carriage of inpatient children developed prior to or during hospital admission. Two children were positive on the first set of screening swabs taken on admission, suggesting that MRSA was acquired elsewhere. An additional two children were positive on the first set of screening swabs but had been inpatients for some time prior to recruitment (7 days and 1 month, respectively), making it impossible to judge whether MRSA carriage developed before or after admission. The remaining two inpatients became positive on their second and third screen, respectively, suggesting that they developed MRSA carriage during this admission.

### MRSA genotypes.

Genotyping data for the 93 MRSA isolates was generated by using a combination of PFGE and MLST (Table 2). A total of five PFGE banding pattern types were identified. The PFGE pattern of most isolates (n = 85, 91%) belonged to a single cluster that matched that of the MRSA ST 834 positive control. MLST of two clinical isolates chosen at random from this group gave a sequence type of ST 834 for both isolates. Three of 93 isolates had a PFGE banding pattern that matched the PFGE banding pattern of the ST 121 positive control and were confirmed by MLST as ST 121. The remaining five isolates belonged to three PFGE banding patterns that did not match either of the positive controls. MLST of all five isolates demonstrate that these belonged to three STs not identified previously in Cambodia (ST 188, ST 45, and ST 9), all of which were isolated from children who came to the OPD. The MRSA isolates cultured from the six inpatients were ST 834 (n = 5, including the two patients with probable nosocomial acquisition) and ST 121 (n = 1, positive on the first swab obtained three days after admission).

Evaluation of the phylogenetic relatedness between the 5 MRSA clones showed that they belonged to four separate lineages: clonal complex (CC) 9 (containing ST 9 and the double locus variant ST 834), CC15 (containing ST 188), CC 45, and CC 121. Three of the five CA-MRSA clones were noted to be predicted founders of a clonal complex (CC 9, CC 45, and CC 121).

### Area of residence for MRSA-colonized outpatients.

**Figure 1.**
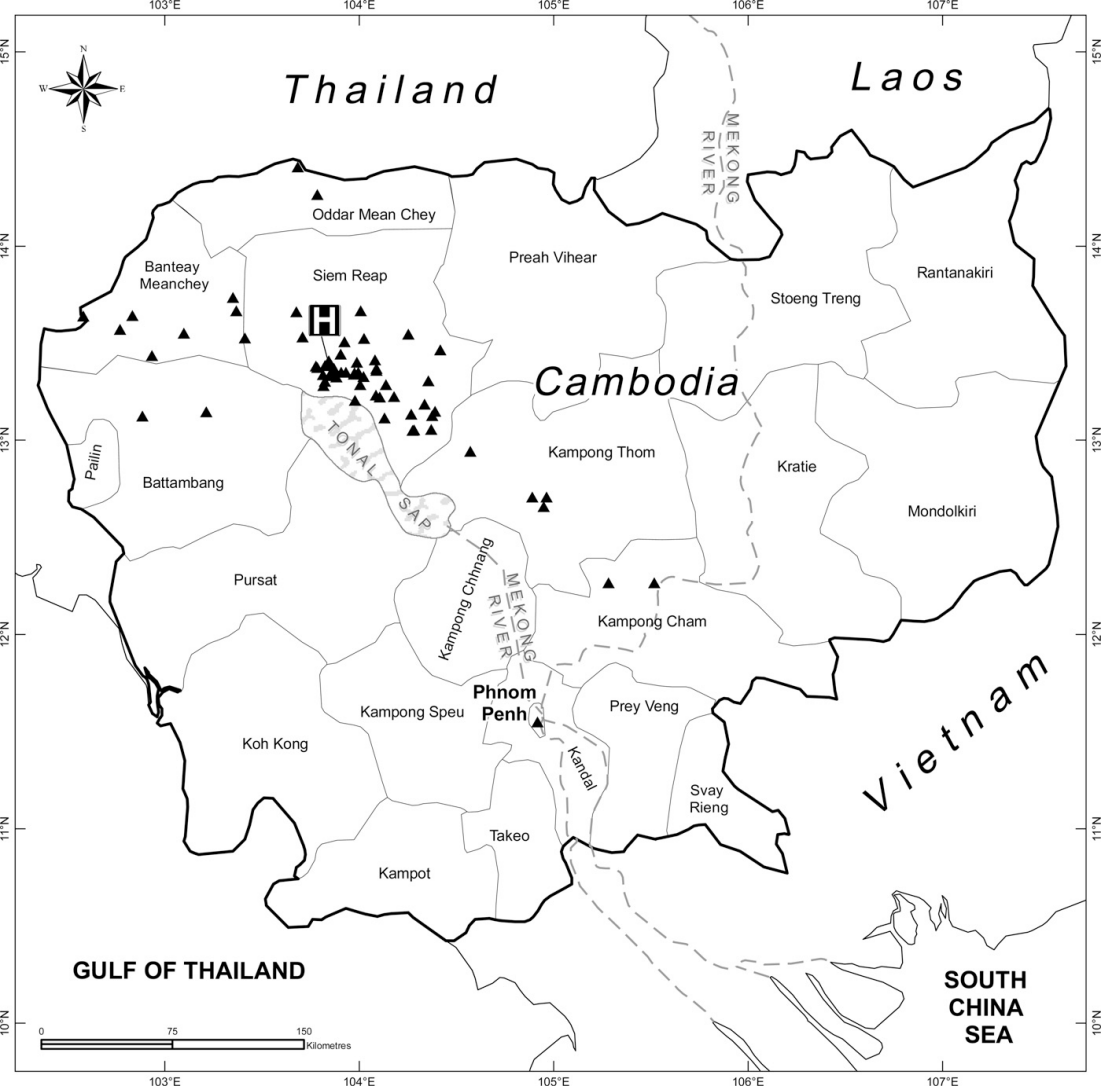
Map of Cambodia showing the place of residence for 87 children who came to the outpatient department of Angkor Hospital for Children, Siem Reap, and were identified as having methicillin-resistant *Staphylococcus aureus* carriage.

Normal place of residence was mapped to examine the geographic distribution of the 87 children who came to the OPD with MRSA (Figure 1). Most (68 of 87, 78%) children lived in Siem Reap Province, and the remainder living in six other provinces. Mapping of the home address for each of the three children carrying ST 188 indicated that they were geographically separated (Phnom Penh, Siem Reap, and a village situated in Kampong Thom Province, respectively). The distance between their addresses ranged from 150 km (Siem Reap to the village in Kampong Thom Province, and the Village in Kampong Thom Province to Phnom Penh) to 300 km (Siem Reap to Phnom Penh).

## Discussion

This study demonstrated that 3.5% of children who came to the AHC during a one-month period in 2008 were colonized with MRSA. This figure may be an under-estimate of true carriage rates because swabbing of children who came to the outpatient department alone received a single nasal swab but no sampling of additional sites of possible colonization was conducted. Acquisition was classified as community-acquired for one-third of cases, but could not be determined for the remainder who had a history of healthcare contact. Healthcare in Cambodia is primarily provided through Ministry of Health community health centers and larger government referral hospitals. No additional insights into place of acquisition could be gained from bacterial genotyping data because nearly all MRSA isolates were ST 834, a clone that most likely has a community origin. The normal place of residence of MRSA carriers encompassed seven provinces in Cambodia. The observation that most MRSA carriers were from Siem Reap Province reflects the overall referral pattern to the AHC. Our data indicate that MRSA has become disseminated throughout northwestern Cambodia, and we predict is likely to have spread across the entire country.

Drivers of CA-MRSA emergence and transmission in Cambodia are unknown, but factors associated with these drivers in remote communities elsewhere are also common in Cambodia. These drivers include uncontrolled sale of over-the-counter antibiotics, sub-standard and over-crowded living accommodation, lack of access to water for bathing, a high frequency of superficial staphylococcal skin sepsis in the community, and frequent scratches/insect bites. Many households in Cambodia will maintain a small number of pigs, but contact with pigs and other animals was not documented during this study. The high use of hospital outpatient departments and community health centers may also be important in this setting, and periodic overcrowding during clinic times and potential barriers faced by healthcare staff to wash their hands between each consultation may also be important factors in this setting. Infection control is poorly developed in most healthcare settings across Cambodia, and the introduction of measures such as the promotion of hand hygiene is affordable and important. Only six health care facilities in Cambodia including the AHC have signed up to the World Health Organization initiative to promote greater awareness of the importance of hand hygiene at the point of care termed ‘SAVE LIVES: Clean Your Hands'.^13^

Most MRSA isolates in this study belonged to ST 834, a clone identified previously as a cause of CA-MRSA infection in the same population. The biological behavior of ST 834, with successful maintenance in the community and nosocomial transmission, indicates that this ST is biologically fit and has the capacity for person-to-person spread. This echoes the history of other CA-MRSA clones such as USA300, which arose in the community but has subsequently disseminated into the hospital setting, blurring its distinction as a community-associated pathogen.^4^,^14^ There are several possible hypotheses for the predominance of ST 834. For example, ST 834 may be more efficient at host-to-host transmission or survival on inanimate objects in the environment, or the introduction may have preceded that of other MRSA clones and therefore have had a greater period of time over which to disseminate.

Antimicrobial drug susceptibility testing of the MRSA isolates identified during this study demonstrated a multidrug-resistant profile, including resistance to several inexpensive oral antibiotics that are in common use in Cambodia. Although the attack rate (the number of infections as a proportion of the number of carriers) for the prevalent MRSA carriage clone (ST 834) is not known, we propose that an overall carriage rate of 3.5% does not warrant changes to empiric prescribing regimens for children with clinical features consistent with *S. aureus* infection in Cambodia. However, it is important that local clinicians be made aware of the possibility of MRSA infection in children with suspected *S. aureus* infection who fail to respond to appropriate antimicrobial drug therapy for MSSA.

Possible options for oral antimicrobial therapy for mild MRSA infection in the community include chloramphenicol or fusidic acid, although the latter drug is not available in Cambodia, but parenteral therapy for invasive MRSA infection is more problematic because vancomycin (and netilmicin) are costly and availability is limited in our setting. Mechanisms of resistance were not investigated in this study but the high rate of rifampicin resistance in the context of the high incidence of tuberculosis in Cambodia^15^ is noteworthy, especially given the low capacity to detect multi-drug resistance in *Mycobacterium tuberculosis* in Cambodia. We did not collect data on resistance in MSSA, and further studies are required to determine rates of antimicrobial resistance in the wider *S. aureus* population.

The finding that there were five independent MRSA clones belonging to four genetic lineages indicates that multiple clones have independently acquired SCC*mec*, the mobile genetic element that carries the gene (*mecA*) responsible for methicillin resistance. It is not clear whether these clones arose in Cambodia or were introduced from elsewhere. MRSA ST 834 has only been isolated in Australia and Cambodia, and MSSA ST 834 has not been identified to date. MRSA ST 9 and MRSA ST 121 have been isolated in China, and MRSA ST 45 and ST 188 are more widely disseminated and have been isolated in Europe; the United States and Australia (ST 45); and China, Malaysia, and Australia (ST 188).

In conclusion, multiple CA-MRSA clones are present in northwestern Cambodia. The MRSA carriage rate in children who came to a single hospital is currently low but further studies are required to track trends over time. The dominant clone (ST 834) carried in the community also predominates in the hospital setting and is associated with nosocomial transmission.

## Figures and Tables

**Table 1:** Summary data for antimicrobial drug susceptibility profiles of 93 methicillin-resistant *Staphylococcus aureus* isolates, Cambodia

Antimicrobial agent	No. (%) resistant isolates
Chloramphenicol	3 (3)
Ciprofloxacin	19 (20)
Clindamycin	70 (75)
Erythromycin	87 (94)
Fusidic acid	0
Gentamicin	14 (15)
Mupirocin	0
Netilmicin	0
Penicillin	93 (100)
Rifampicin	84 (90)
Tetracycline	87 (94)
Trimethoprim/sulfamethoxazole	84 (90)
Vancomycin	0

**Table 2:** Genotype based on PFGE and MLST for 93 methicillin-resistant *Staphylococcus aureus* isolates, Cambodia*

No (%) isolates	PFGE banding pattern type	Sequence type
85 (91)	AHC 1	ST 834
3 (3)	AHC 2	ST 121
3 (3)	AHC 3	ST 188
1 (1)	AHC 4	ST 45
1 (1)	AHC 5	ST 9

* PFGE = pulsed-field gel electrophoresis; MLST = multilocus sequence typing.
